# Foraging *Macrotermes natalensis* Fungus-Growing Termites Avoid a Mycopathogen but Not an Entomopathogen

**DOI:** 10.3390/insects10070185

**Published:** 2019-06-26

**Authors:** Kasun H. Bodawatta, Michael Poulsen, Nick Bos

**Affiliations:** 1 Section for Ecology and Evolution, Department of Biology, University of Copenhagen, 2100 Copenhagen East, Denmark; 2 Natural History Museum of Denmark, University of Copenhagen, 2100 Copenhagen East, Denmark

**Keywords:** *Beauveria*, defence, social immunity, *Termitomyces*, *Trichoderma*

## Abstract

Fungus-growing termites have to defend both themselves and their monoculture fungal cultivars from antagonistic microbes. One of the ways that pathogens can enter the termite colony is on the plant substrate that is collected by termite foragers. In order to understand whether foragers avoid substrate infected with antagonists, we offered sub-colonies of *Macrotermes natalensis* a choice between food exposed to either a mycopathogenic or an entomopathogenic fungus, and control food. Workers did not show any preference between entomopathogen-exposed and control substrate, but significantly avoided the mycopathogen-exposed substrate. This suggests that the behaviour of foraging workers is more strongly influenced by pathogens affecting their crop than those posing risks to the termite workers themselves.

## 1. Introduction

The evolutionary success of a social lifestyle is evident in many insect groups, such as ants, termites, bees, and wasps, through their diversity, cosmopolitan distribution and ecological impact [1,2,3,4,5]. Although there are many advantages [6,7], sociality also poses challenges [8,9], including increased vulnerability to infections from antagonistic microbes due to the high densities and typically low genetic diversity between colony members [8,10]. Coping with these changes in disease pressures involved co-option of pre-existing defences (e.g., [11,12,13,14,15]) in a social context, and the evolution of social immunity mechanisms [8,16], including behavioural modifications [11,17,18,19], soldier defences [20,21], utilization of bacterial symbionts [22] and development of extraordinary sensory abilities to detect antagonists [23].

Within the ants (Hymenoptera, Formicidae) and termites (Blattodea, Termitidae), two clades have independently taken the social life style to the next level through the evolution of farming their own fungal food [24]. The domestication of fungal symbionts has contributed to the successful radiation of fungus-growing ants (Formicidae, Myrmicinae, Attini) in the New World [25] and fungus-growing termites (Termitidae, Macrotermitinae) in the Old World [26], where they play major roles in plant biomass decomposition and nutrient cycling [4,5,27,28,29,30]. These farming insects have long-lived colonies and keep their fungal cultivars in monoculture [31,32], which, along with their social life style, may make them particularly vulnerable to infections [12,33]. While ant fungal gardens get frequent invasions by the fungal pathogen *Escovopsis* [34], fungus-growing termites appear to manage to keep their fungal cultivar disease free [35,36,37]. In the past decades, several studies have focused on understanding the mechanisms of how both these insect hosts manage to keep their fungal gardens protected from pathogens [18,38,39,40,41,42,43,44,45], but most research has focused on fungus-growing ants [39,40,41,42,43,46].

In fungus-growing termites, the removal of workers and soldiers from fungal gardens leads to the emergence of antagonistic and weedy fungi, such as *Pseudoxylaria* (Ascomycota, Xylariaceae) and *Trichoderma* (Ascomycota, Hypocreaceae), emphasizing the importance of the termites for keeping the fungal cultivar healthy [35,37]. Termites employ a number of defences, such as burying weedy fungi to suppress their growth [18,47], and possible utilisation of symbionts such as *Bacillus* sp. against antagonistic fungi [38]. Foraging on material exposed to entomopathogenic (pathogens of the termites) and mycopathogenic (pathogens of the fungus) fungi could threaten colony health. However, although termite workers possess the ability to recognize entomopathogenic fungi using olfactory cues [23], we do not know whether foraging workers avoid forage material infected with pathogenic fungi. To better understand the role of foraging workers in keeping the colony healthy, we assessed whether foragers of the fungus-growing termite *Macrotermes natalensis* Haviland avoid entomopathogenic and mycopathogenic fungi. We hypothesised that workers should recognize both types of pathogens and reduce their foraging activity in chambers containing infected material in order to avoid introducing potential threats to themselves or to their mutualistic fungus. 

## 2. Materials and Methods 

### 2.1. Field Sites and Termite Nest Collection

*M. natalensis* major workers, minor soldiers, and fungus gardens (combs) were collected from two nests (colony Mn186 and Mn190) from Gauteng (S 25° 44.600’, E 28° 15.648’) and one nest (colony Mn187) from Limpopo (S 24° 40.434’, E 28° 48.275’) in South Africa in January 2018. Termites and fungal gardens were kept in a climate-controlled room (26 °C) for a maximum of one day before the experiment was carried out. Pieces of decaying wood were collected near termite nests to be used as foraging material in the experimental setups, and these were autoclaved for 20 min. at 121 °C to kill environmental microbes. 

### 2.2. Fungal Pathogens

We used *Beauveria bassiana* MacLeod, a generalist entomopathogenic fungus [48], and *Trichoderma* sp., a generalist mycopathogen of the fungal garden [36], to investigate forager responses to fungus exposed foraging material. Both strains had been isolated from fungus-growing termite nests in 2013 (*B. bassiana* isolate #122 from *M. natalensis* colony Mn134; *Trichoderma* sp. isolate also from a *M. natalensis* colony). The fungi were grown on Potato Dextrose Agar (PDA) medium (32.1 g PDA, 4 g agar, 800 mL distilled water) for ~14 days to ensure that conidia were produced. Conidia were collected immediately before the experiment and were added to 0.1% Tween80 in sterile water to create suspensions that were subsequently diluted to a concentration of 10^6^ conidia per mL [49].

### 2.3. Behavioural Setup

**Figure 1:**
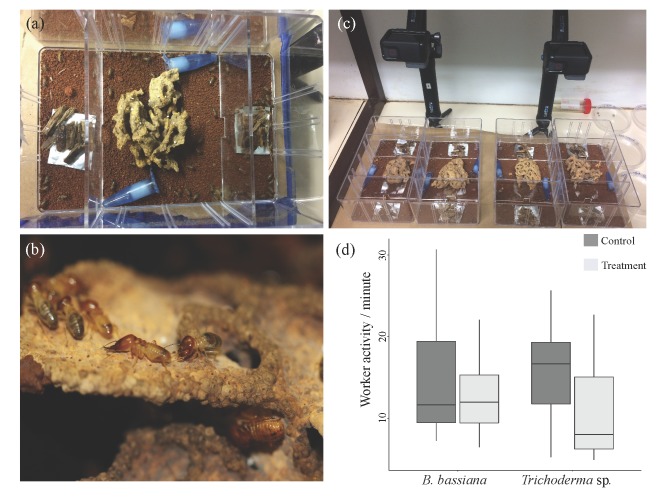
(**a**) The sub-colony setup, consisting of a central chamber with the fungus garden and two foraging chambers (left and right). In each sub-colony, one of the two foraging chambers contained foraging material treated with either *B. bassiana* or *Trichoderma* sp. (photo by K.H.B). (**b**) *M. natalensis* major termite workers and soldiers on a fungus comb (photo by M.P.). (**c**) The experimental set up with two replicates and two treatments (*B. bassiana* and *Trichoderma* sp.) (photo by K.H.B). (**d**) Boxplot demonstrating worker activity per minute in foraging chambers treated with *B. bassiana* or *Trichoderma* sp., and control chambers. Horizontal lines denote the median, whiskers extend from the first and third quartile and any data points outside of this range would be denoted as outliers and plotted individually, however, no such points existed in the dataset (Table S1).

For each colony, four sub-colonies were created in transparent plastic boxes (17 × 10.5 × 6 cm), and a thin layer of autoclaved soil (collected near the original mound) was added to the bottom. The soil retained some moisture after autoclaving and was not moistened further. The boxes were divided into three chambers using plastic dividers: a central chamber and two foraging chambers, one on each side of the central chamber (Figure 1a). The chambers were connected to each other through a 1 cm^2^ entrance opening in the centre of each divider. Two 1.5 mL Eppendorf^®^ tubes with water and a cotton wool plug were added to the central chamber to maintain high humidity. One piece of fungus comb (~64 cm^3^), one hundred major workers, and five minor soldiers were introduced to the central compartment six hours prior to the start of the experiment (Figure 1b), and the openings to the side chambers were closed. The five minor soldiers were added as it has been shown previously to help keep sub-colonies healthy [50]. 

Ten minutes before the start of the experiment, 1 g of autoclaved wood was provided on a piece of aluminium foil to each foraging chamber. The four sub-colonies per colony were then randomly divided in two treatments (resulting in two technical replicates per colony). In the first treatment, the wood in one foraging chamber was treated with *B. bassiana* through inoculating 300 µL conidia suspension directly on the wood using a pipette, while in the second treatment, the wood was treated with *Trichoderma* sp. conidia suspension. In both treatments, the wood in the other foraging chamber was treated with 0.1% Tween80 solution as a control. After ten minutes, the entrances from the central chamber to the foraging chambers were opened, and GoPro^®^ Hero 5 Black cameras were used under red light to record worker behaviour for a total of ca. two hours (Figure 1c). Recording of all sub colonies per colony was done simultaneously. 

### 2.4. Analysis of Behaviours

All video recordings were viewed by the same person. For each colony/treatment combination we acquired a total of ca. two hours of video recordings. However, these were recorded in several video clips that were roughly 18 minutes in length each due to the nature of the cameras used. We did not analyse the first video recorded (the first ca. 18 minutes) of each sub-colony in order to avoid potential behavioural biases due to handling during inoculations and to allow the termites to scout the newly accessible locations in their nest box, resulting in 5–6 videos per colony/treatment combination (except for replicates 186_C and 186_D, which have 4 videos). Movement to and from each side of the box (fungus-treated vs control) was defined as the sum of the number of in- and outgoing individuals per video. The observed movement value was then divided by the length of the video in minutes to achieve a measure of worker activity per minute (Table S1). This resulted in a total of 60 samples, with 5–6 samples per replicate.

Worker activity was the dependent variable in a linear model, with treatment (*B. bassiana* vs *Trichoderma* sp.) and side (control vs treated side of the box) as fixed effects. Replicate, nested within colony, was added as a fixed effect to account for pseudo-replication. Adding these as random effects would not be proper here, due to the low number of colonies (as a minimum of five levels is advised for random effects [51]). The model was validated using diagnostic plots, which identified one data point as a potential outlier, so we ran the model both with and without this data point. This did, however, not change the results (adjusted R^2^ = 0.79 and 0.80 for the full and the reduced model, respectively), and we report the full model (Table S2). The analysis was carried out in RStudio (R version 3.5.1), and the package ggplot2 was used to create figures [52,53].

## 3. Results

Worker activity in the *B. bassiana* did not significantly differ from those in the *Trichoderma* sp. treatment (lm, treatment, *t* = 0.38, *p* = 0.71). Indeed, the mean ± SE total number of foragers per minute was 13.48 ± 0.72 for *B. bassiana* and 13.04 ± 0.78 for *Trichoderma* sp. There was a significant interaction effect of treatment and box side, where in the case of *Trichoderma* sp., workers preferred the side of the box that contained control foraging material, while in the case of *B. bassiana*, workers visited both sides of the box equally (lm, treatment x side, *t* = -3.45, *p* < 0.001, Figure 1d).

## 4. Discussion

Fungus-growing termites have to protect both themselves and their fungal crop from potential pathogens present in the substrate they forage on. We hypothesised that termites would avoid contaminated substrates, irrespective of whether the pathogen would target the termites themselves or their fungus garden. However, we found that workers avoided the mycopathogenic fungus (*Trichoderma* sp.), but not the entomopathogen (*B. bassiana*). This may imply that termites are more cautious towards fungus garden contaminants, and are adapted to respond accordingly. However, exposure to both entomopathogenic and mycopathogenic fungi simultaneously in a more direct comparison would have provided more insights to possible differential avoidance behaviour in this termite species. 

Termites possess mechanisms to counteract both entomopathogenic [11,14,16,21,54] and mycopathogenic [18,38] fungi, but our findings suggest that they rely more on avoidance behaviour toward mycopathogens and presumably employ other defences, such as their innate immune system, toward entomopathogens. Fungus-growing termites have the ability to recognize *B. bassiana* through olfactory cues [23]. Therefore, although they likely detected the presence of *B. bassiana* in our experiment, this did not translate to avoidance, deviating from the results of previous studies [23,55,56], which found that termite workers were repelled by all *B. bassiana* strains tested. However, unlike those studies, our experiment was conducted in the context of foraging. The lack of avoidance could thus potentially be explained by the fact that *B. bassiana* is a widespread, facultative, generalist entomopathogen [48], and avoidance could be too costly during foraging. Termites are also equipped with many innate [12,15] and social immune [10,11,21,57] mechanisms against these common entomopathogens and the cost of avoidance of substrates may thus be higher than that of an immune response. Alternatively, the termites may have been able to identify the virulence level of the *B. bassiana* strain used in this experiment and was able to recognize it as a less virulent strain [23,55,57]. Although we have not assessed the virulence of this particular strain, we deem this hypothesis to be less likely, at least in a non-foraging context, as even low-virulence strains elicit an avoidance reaction [23,55,57].

If termites indeed respond differentially to threats to themselves vs. fungus garden antagonists, this could imply that costs of infected workers versus infected fungus comb differ and thus select for prioritisation of the health of the fungal cultivar. Individual termite infections are likely to be localized, allowing for infected workers to be isolated and treated (e.g., with soldier secretions [21], cannibalised or buried [57]). Infected individuals may also be able to self-exclude from the colony, which has to our knowledge not been documented in termites, but has been found in other social insects, such as ants and honey bees [19,58,59]. The cost of losing individual termites due to entomopathogen infections is unlikely to pose a big threat to the overall wellbeing of the colony. In contrast, the fungal garden is a continuous mass within which infections can spread and where isolation of infected areas may prove costly and more difficult to contain. In fungus-growing ants, weeding and grooming of the fungus garden are effective [60], but nevertheless often lead to incomplete pathogen removal, with colonies consequently experiencing sub-lethal endemic levels of infection [34], which likely impacts colony growth and thus ultimately fitness. A similar scenario is conceivable in fungus-growing termites, and may select for particularly careful avoidance of mycopathogen-infested substrates. 

The results of this study raise the question whether termite workers prioritize the health of their fungal cultivar over their own health. This is an important question to answer in order to better understand the evolutionary stability of this insect-fungus symbiosis. Although we did not test this directly, it appears that workers are more cautious about bringing in mycopathogenic fungi compared to entomopathogens to their colony. Therefore, to test whether workers prioritize the health of their fungus cultivar over themselves, next to assessing the virulence of the *B. bassiana* strain to the termites, it would be interesting and important to evaluate behaviours of foragers exposed to entomopathogenic and mycopathogenic fungi simultaneously. 

## 5. Conclusions

To date, little research has been done to explore the defence mechanisms employed by fungus-growing termites to keep their monoculture fungal cultivars disease free [18,37,38,47,61]. We provide the first indication of a behavioural response by foraging workers toward infected and non-infected forage material, where workers reduce their activity towards a pathogen of the fungal garden. Foraging workers may thus act as the first line of defence in keeping fungus gardens free from infections through identifying and avoiding potential fungal garden pathogens. 

## Supplementary Materials

The following are available online at https://www.mdpi.com/2075-4450/10/7/185/s1, Table S1: Number of foraging workers visited the two sides (fungal treated vs control) of each sub colony under two treatments (*B. bassiana* vs *Trichoderma* sp.) in each video recording. Table S2: R script used to conduct the statistical analysis.
